# Physical activity, sleep quality and life satisfaction in adolescents: A cross-sectional survey study

**DOI:** 10.3389/fpubh.2022.1010194

**Published:** 2022-12-20

**Authors:** Yunhua Cao, Zhen Yang, Yongbo Yu, Xin Huang

**Affiliations:** ^1^Department of Physical Education, North China Electric Power University, Beijing, China; ^2^Physical Activity for Health Research Centre, Moray House School of Education and Sport, University of Edinburgh, Edinburgh, United Kingdom; ^3^School of Physical Education and Sport Training, Shanghai University of Sport, Shanghai, China; ^4^School of Physical Education and Sports Science, South China Normal University, Guangzhou, China

**Keywords:** sleep quality, life satisfaction, teenagers, cross-sectional studies, mental health

## Abstract

**Background:**

Physical activity, sleep is an important component of adolescents' 24-h movement behavior, and life satisfaction predicts adolescent physical and mental health. However, few studies have explored the relationship between the two variables among Chinese high school students. Consequently, this study aimed to explore the relationship between sleep quality and life satisfaction among Chinese high school students.

**Methods:**

This cross-sectional study was conducted in October 2020 in five high schools in Xuchang City, China. High school students from senior 1 to senior 3 were asked to fill up a questionnaire of demographic characteristics and validated screen instructions for sleep quality (Pittsburgh Sleep Quality Index) and life satisfaction (Satisfaction with Life Scale). The multivariate regression model was applied to explore the association between sleep quality and life satisfaction.

**Results:**

A total of 1127 individuals (51.1% males) participated in this survey, and the prevalence of poor sleep quality was 42.3%. The mean score on the Satisfaction with Life Scale was 16.72 ± 5.67, according to the corresponding scoring criteria, with 15–19 being generally dissatisfied. After controlling for demographic variables, the contribution of subjective sleep quality (β = – 0.181, *p* < 0.01), sleep duration (β = – 0.080, *p* < 0.01) and daytime dysfunction (β = – 0.311, *p* < 0.01) to life satisfaction of high school students increased to 23.2%, indicating that subjective sleep quality, sleep duration, and daytime dysfunction were 22.9% predictive of life satisfaction among high school students. And also, physical activity on schooldays and weekend days were associated with better sleep quality and higher life satisfaction (*p* < 0.05).

**Conclusions:**

Among Chinese high school students, the prevalence of sleep problems was high, and the majority of students held moderate life satisfaction. Sleep quality was positively correlated with life satisfaction among Chinese high school students, with better sleep quality resulting in higher life satisfaction among students.

## Introduction

Sleep is an important component of the 24-h movement behaviors of adolescents, while poor sleep quality is prevalent among this population (1–6). There has been a significant decline in sleep quality among adolescents (7), while this decline has been exacerbated by the COVID-19 outbreak (8–12). Not only the pandemic, but also physiological, psychological, demographic, geographical, and socioeconomic factors influence the prevalence of sleep quality in adolescents (13–15). Several studies from China proposed that the prevalence of poor sleep quality among high school students ranged from 8.54 to 41.9% (16, 17). Moreover, the prevalence of poor sleep quality was the highest among senior high school students compared to students in primary school, junior high school, and college (18). Studies from Malaysia and Italy reported similar prevalence rates of 24.0 and 18%, respectively (19, 20). However, the prevalence of poor sleep quality among high school students in Spain, Turkey and Sweden is close to or over 60%, with a prevalence of 76% among Swedish girls (21–23). Poor sleep quality among adolescents has become a serious public health problem worldwide, with many negative consequences (24–26). Numerous observational studies showed an association between poorer sleep quality and poorer learning capacity and academic performance (27, 28). Moreover, high school students with poorer sleep quality were more likely to engage in school bullying (29). In terms of addictive behaviors, a large number of studies reported the relationship between poor sleep quality and nicotine dependence, internet addiction, and substance abuse (21, 22, 30, 31). Additionally, poor sleep quality was a risk factor for recurrent aphthous ulcers and primary nocturnal enuresis (32, 33). In terms of mental health, poor sleep quality has been demonstrated as a risk factor for poor mental health conditions, such as depression, anxiety and lower subjective well-being (8, 34–37). Furthermore, several cross-sectional studies reported that high school students with lower sleep quality were more likely to have suicidal ideation and carried out non-suicidal self-injury (34, 38, 39).

Life satisfaction is an individual's general perception of their life situation over a period of time (40). Life satisfaction is an important parameter reflecting an individual's quality of life, and a very important component of psychological well-being (26, 41–44). Previous research has shown that life satisfaction is not only influenced by internalized emotions such as depression and anxiety (45), but also by modifiable lifestyle behaviors (46). Furthermore, among adolescents, life satisfaction predicts their level of mental health and social problem-solving skills (47–49).

Among adolescents, a few studies have explored the relationship between modifiable lifestyle behavior and life satisfaction. Schmiedeberg et al. found that participation in leisure activities (parties, sports, holidays) was positively associated with life satisfaction among German teenagers and adults, while Internet and TV consumption were negatively associated with life satisfaction (50). In another survey of 245 high school students, it was reported that low physical activity and nonparticipation in sports teams were associated with lower life satisfaction, where the number of siblings in the family also has an effect (51). A cross-sectional study from China found that prolonged screen time and lower levels of physical activity were associated with lower life satisfaction among Chinese junior high school students (52). A large random sample longitudinal study of German adolescents and adults (15–41 years old during the observation period) by Schmiedeberg et al. tested the effects of five leisure activities (sports, vacation, meeting friends, internet use, and watching TV) on respondents' life satisfaction and found that some leisure activities (partying, sports, vacation) had a positive effect on life satisfaction, and while Internet and TV consumption had a negative effect on life satisfaction (50). Zullig et al. administered physical activity, life satisfaction, and self-evaluation questionnaires to 245 7th and 8th grade students and found that females who had not exercised vigorously in the past 7 days had significantly lower life satisfaction and those who did not participate in sports teams had satisfaction was lower, and regression analysis showed that physical activity increased life satisfaction among secondary school students, and that secondary school students with more sports participation had higher life satisfaction than those with less sports participation (51). In addition to physical activity and sedentary behavior, two modifiable lifestyle behaviors, previous studies have also explored the relationship between sleep quality and life satisfaction among adolescents. Ness et al. proposed that among 701 Norwegian university students, good sleep quality, long sleep duration, and stability in sleep and wake times were associated with higher life satisfaction (53). While the correlation between greater sleep quality and higher life satisfaction was also found among university students in Malaysia (54). Nevertheless, no studies have investigated the relationship between sleep quality and life satisfaction in this large population of Chinese adolescents. Previous studies have only investigated the relationship between sleep quality and life satisfaction among Chinese university students and older adults (55, 56), whereas studies with a representative sample of Chinese adolescents are scarce.

Therefore, this study will investigate the prevalence of sleep quality and life satisfaction among Chinese high school students during the COVID-19 pandemic period, and explore the relationship between sleep quality and life satisfaction in order to provide a theoretical basis for improving high school students' life satisfaction through improving sleep quality and contributing to their mental health.

## Methods

### Study design

A cross-sectional design was adopted for this study, which was conducted in October 2020 in Xuchang City, China. Stratified random sampling was utilized to sample students from five high schools in Xuchang City. Demographic information, quality of sleep and satisfaction with life by questionnaire. After calculating the sample size by G power, two classes of students from grades 1–3 were selected in each high school for the survey. This study was conducted by paper-based questionnaires. To ensure the return rate of the questionnaire, it was uniformly explained by the class directors and distributed with the class during the students' lunch break, and students were asked to fill in and return it independently. This study is a cross-sectional study. The sleep quality and life satisfaction of high school students require a two-sided test. A is 0.05. According to the relevant literature, the prevalence of indicators is 13%, and the allowable error d is 0.02. Calculate the sample size according to the following formula. The available sample size is *n* = 1087. A total of 1270 students agreed and participated in this research survey. 1127 valid questionnaires were obtained after excluding invalid questionnaires with missing important information, with a response rate of 88.74%. The design of the present study was in accordance with the Declaration of Helsinki, and signed informed consent was obtained from the participants prior to the start of the study. This study was approved by the Institutional Review Board (IRB) of the Shanghai University of Sport (SUS), and permission to carry out the study was obtained from teachers and principals of participating schools.

### Measurement

#### Sleep quality

The Pittsburgh Sleep Quality Index (PSQI) was utilized in this study to measure the sleep quality of high school students (57). This scale comprises 19 self-reported items and 5 other-reported items. The 18 self-reported items are scored on a total of seven components for sleep latency, sleep duration, habitual sleep efficiency, sleep disturbances, subjective sleep quality, use of sleep medication, and daytime function in the past month. The 7 components have their corresponding scoring conventions, which are converted into a corresponding score (0–3) and added together to give a final total score (0–21) for the overall sleep quality, with higher scores indicating poorer sleep quality. The PSQI classifies sleep quality into three categories, with a score of < 3 indicating good sleep quality, 3 to 7 indicating moderate sleep quality and >8 indicating poor sleep quality (57). The Chinese version of PSQI has been reported to have acceptable reliability and validity in the Chinese population (58).

#### Life satisfaction

Life satisfaction in this study was measured using the Satisfaction with Life Scale (SWLS) developed by Diener (59). This scale consists of 5 items on a 7-point Likert scale ranging from 1 for very dissatisfied to 7 for very satisfied. The scores for each item are added up, with higher scores indicating higher life satisfaction. Life satisfaction scores can be divided into seven levels, with 5–9 being very dissatisfied, 10–14 being dissatisfied, 15–19 being generally dissatisfied, 20 being neutral, 21–25 being generally satisfied, 26–30 being satisfied and 31–35 being very satisfied (59). The Chinese version of SWLS has been demonstrated to have acceptable validity and reliability in the Chinese population (60).

#### Covariates

Sociodemographic characteristics, which consisted of grade (senior 1, senior 2, and senior 3), gender (male, female), residence (urban, rural area), and whether they are an only child (yes or no), were identified as covariates for further analysis.

### Statistical analysis

Excel 2016 was utilized for data entry and storage, while all the statistical analysis was performed using SPSS 20.0. The demographic characteristics, sleep quality and life satisfaction were reported using a descriptive approach. One-way ANOVA was used to analyze differences in demographic variables between sleep quality and life satisfaction among high school students, while Person's correlation analysis was used to examine the correlation between sleep quality and life satisfaction. continuous variables were expressed as mean and standard deviation (mean ± standard deviation), and categorical variables were expressed as numbers (n) and percentages (%). Multiple linear regression analysis was performed to analyze the relationship between sleep quality and life satisfaction, controlling for demographic variables, and statistical significance was established at the *p* < 0.05 level.

## Results

### Demographic characteristics

A total of 1127 valid questionnaires with a balanced gender distribution were obtained, of which 51.1% were male. There was also a balanced distribution of participants by grade, all between 30 and 40 per cent, with the highest number of students in the senior 1 grade (37.4%). Most of the students lived in rural areas (64.0%) and the majority were the only children (85.0%) in their families (see Table 1).

**Table 1:** Demographic characteristics (*n* = 1127).

	**N**	**%**
**Gender**		
Male	576	51.1
Female	551	48.9
**Grade**		
Senior 1	421	37.4
Senior 2	355	31.5
Senior 3	351	31.1
**Residence**		
Urban	406	36.0
Rural area	721	64.0
**Whether only child**		
Yes	169	15.0
No	958	85.0

### Prevalence of sleep quality and life satisfaction

Prevalence of sleep quality and life satisfaction among high school students in Table 2. 43.7% of high school students had moderate life satisfaction, followed by participants who were generally dissatisfied (26.0%) and the lowest number of very satisfied (1.2%). In terms of sleep quality, half of the total, had moderate sleep quality (50.0%), followed by those with poor sleep quality (42.3%) and very few with good sleep quality (7.7%).

**Table 2:** Prevalence of sleep quality and life satisfaction (*n* = 1127).

	**N**	**%**
**Life satisfaction**		
Very dissatisfied	47	4.2
Dissatisfied	114	10.1
Generally dissatisfied	293	26.0
Neutral	492	43.7
Generally satisfied	120	10.6
Satisfied	48	4.3
Very satisfied	13	1.2
**Sleep quality**		
Good sleep quality	87	7.7
Moderate sleep quality	563	50.0
Poor sleep quality	477	42.3

### Association between sleep quality and life satisfaction

In order to examine the relationship between sleep quality and life satisfaction, regression analysis was conducted with life satisfaction as the dependent variable, and gender, grade, residence, and whether only child was treated as independent variables to construct model 1. The independent variables in model 1 were treated as controlled variables, and the sleep quality was treated as the independent variable to construct model 2. The results of regression analysis were clearly presented in Table 3. In Model 1 (Correlation coefficient = 0.083, Coefficient of determination = 0.007, Adjusted coefficient of determination = 0.003), grade (β = – 0.075, *p* < 0.05) was a statistically significant predictor of life satisfaction for high school students, with a contribution of 0.3%. After controlling for demographic variables (Correlation coefficient = 0.489, Coefficient of determination = 0.239, Adjusted coefficient of determination = 0.232), the contribution of subjective sleep quality (β = – 0.181, *p* < 0.01), sleep duration (β = – 0.080, *p* < 0.01) and daytime dysfunction (β = – 0.311, *p* < 0.01) to the life satisfaction of high school students increased to 23.2%, indicating that subjective sleep quality, sleep duration, and daytime dysfunction were 22.9% predictive of life satisfaction among high school students.

**Table 3:** Regression results of sleep quality and life satisfaction (*n* = 1127).

	**Life satisfaction**					
	**Model 1**			**Model 2**		
	**B**	**SEx**	**β**	**B**	**SEx**	**β**
Gender	– 0.197	0.337	– 0.017	– 0.005	0.302	0.000
Grade	– 0.517	0.204	– 0.075*	– 0.080	0.185	– 0.012
Residence	– 0.103	0.365	– 0.009	– 0.420	0.323	– 0.036
Whether only child	0.480	0.491	0.030	0.277	0.436	0.017
Subjective sleep quality				– 1.297	0.236	– 0.181**
Sleep duration				– 0.596	0.226	– 0.080**
Daytime function				– 2.020	0.191	– 0.311**

R, correlation coefficient; R^2^, coefficient of determination; R^2^adj, adjusted coefficient of determination; B, unstandardised regression coefficient; Sex, standard error; β, standard regression coefficient; *represents *p* < 0.05; **represents *p* < 0.01.

### Association between physical activity and life satisfaction

In order to examine the relationship between physical activity and life satisfaction, regression analysis was conducted with life satisfaction as the dependent variable, and gender, grade, residence, and whether only child was treated as independent variables to construct model 2. The independent variables in model 2 were treated as controlled variables, and physical activity was treated as the independent variable to construct model 2. The results of regression analysis were clearly presented in Table 4. In model 1, the correlation coefficient, the coefficient of determination, and the adjusted coefficient of determination is 0.083, 0.007, and 0.003, respectively. In model 2, the correlation coefficient, the coefficient of determination, and the adjusted coefficient of determination is 0.217, 0.047, and 0.042, respectively. After controlling for demographic variables, the results suggested that physical activity regardless of weekdays and weekend days were associated with better sleep quality.

**Table 4:** Regression results of physical activity and life satisfaction (*n* = 1127).

	**Life satisfaction**					
	**Model 1**			**Model 2**		
	**B**	**SEx**	**β**	**B**	**SEx**	**β**
Gender	– 0.197	0.337	– 0.017	0.141	0.334	0.012
Grade	– 0.517	0.204	– 0.075*	– 0.475	0.200	– 0.069*
Residence	– 0.103	0.365	– 0.009	– 0.037	0.358	– 0.003
Whether only child	0.480	0.491	0.030	0.625	0.489	0.039
Schooldays physical activity				0.392	0.129	0.113**
Weekend days physical activity				0.915	0.303	0.113**

R, correlation coefficient; R^2^, coefficient of determination; R^2^adj, adjusted coefficient of determination; B, unstandardised regression coefficient; Sex, standard error; β, standard regression coefficient; *represents *p* < 0.05; **represents *p* < 0.01.

## Discussion

In our knowledge, this study is the first study with a representative sample to investigate the prevalence and relationship of sleep quality and life satisfaction among Chinese adolescent in high school. This present study found that the prevalence of sleep problems was high among adolescent high school students in China, and that the majority of students held moderate life satisfaction. Sleep quality was positively correlated with life satisfaction among Chinese high school students, with better sleep quality resulting in higher life satisfaction among students. Furthermore, there was a positive association between higher sleep quality and higher life satisfaction, while sleep duration and daytime dysfunction significantly predicted life satisfaction among Chinese high school students.

The prevalence of sleep problems in this study was 42.3%, which is consistent with a meta-analysis on sleep quality among adolescent students in China, which mentioned a 13.9–44.8% detection rate of sleep disorders among college and high school students (61). The quality of sleep of Chinese high school students is influenced by many personal and social factors, such as family relationships, their own experiences, and academic stress (62). Especially in high school, adolescents must face huge changes in adolescence and the enormous pressure of the entrance exam of higher education at the same time, which can easily lead to anxiety, depression, and other psychological problems, and also reduce sleep quality. Furthermore, more high school students had moderate to poor sleep quality than good sleep quality, which is consistent with the founding of the study by Zhao et al. (63). Compared to other studies in the context of the COVID-19 pandemic in China, the prevalence of poorer sleep quality in this study was higher (8, 64). In cross-sectional studies, one possible explanation for the differences in results across studies is cohort effect (65, 66). Moreover, all participants in the present study were high school students, whereas participants in the previous study included both middle school and high school students (8, 64). High school students had poorer sleep quality than middle school students (18, 67), thus differences in sampling may contribute to differences in the prevalence of sleep quality between studies.

The SWLS scores of the adolescents in this study were lower than those of the previous study, which may be due to the difference in the age of the sample size (68). In the previous study, the participants were all undergraduate students from a medical science university (68), while the participants in the present study were all high school students. This group of high school students is under immense pressure to progress to higher education, whereas undergraduates are not under pressure to progress and have more freedom and time. Although undergraduate medical students also experience high levels of academic stress, it is not possible to analyze this qualitatively or quantitatively in relation to the stress of high school students taking university entrance examinations. Therefore, different ages, different social life circumstances and different academic stress may have contributed to this paradox. The prevalence of dissatisfaction with life in this study is similar to another cross-sectional survey of high school students in China, which may corroborate that different ages, social life circumstances, and academic pressures can influence adolescents' life satisfaction (69). In the context of the COVID-19 pandemic, no study has investigated the prevalence of subjective well-being among Chinese adolescents. Xiao et al. investigated the subjective well-being of Chinese university students and found that mean life satisfaction was 20.51 (70), representing neutrality according to the SWLS scoring scale. The highest percentage of subjective well-being status in the present study was also neutrality, which is similar to the results of previous studies.

In terms of the relationship between sleep quality and life satisfaction, this study found a negative relationship between total sleep quality score and life satisfaction, suggesting that the better the sleep quality, the higher the life satisfaction, which is consistent with existing studies (71). This may be due to the fact that sleep is a basic physiological need of the human body, and good sleep quality can ensure that high school students maintain a better mental state in their daily study and life. At the same time, the influence of physical activity on the life satisfaction of high school students should also be considered [33]. The results show that there is a positive correlation between school day PA, weekend PA and life satisfaction of high school students, adolescents with higher levels of physical activity have higher life satisfaction, and through the consumption of energy and catharsis of emotions during participation in physical activity, not only can improve the physical problems of high school students, but also reduce the generation of bad emotions and enhance the positive perception of life status, thus increasing the life satisfaction of high school students. Students who have good sleep quality are more confident in life and can better enjoy the various pleasures in life, with less negative emotions and more positive emotions leading to increased life satisfaction. The regression results of this study showed that the total score of PSQI was a significant negative predictor of life satisfaction for high school students, which is consistent with existing studies (72, 73). High school students who sleep better will be able to recover and recharge their strength and energy, show more confidence in their daytime life and study, and have a more positive attitude toward life and study, while students who do not sleep well will be depressed, less positive, and less motivated to do things, resulting in less satisfaction in life. In further, lower sleep duration was also a predictor of poorer life satisfaction, which is consistent with the results of previous studies on the same age group (73). Lemola et al. found that short sleep duration (< 6 h per day) and long sleep duration (>9 h per day) were associated with lower life satisfaction (73). Therefore, based on the findings of this study, we suggest that schools and families should pay more attention to the sleep of high school students in their daily lives. The causes of reduced life satisfaction come not only from academic stress, but also from a range of sleep problems that do not easily attract attention. Schools need to set a scientific schedule for students' work and rest, not too long or too short. It is important to ensure that high school students get enough sleep or reduce the amount of homework as appropriate, thus to reduce the phenomenon of high school students writing homework after school until midnight or even early in the morning. Students' lunch breaks can also be appropriately increased to ensure the efficiency of their daytime studies, so as to improve the quality of their sleep and thus increase their life satisfaction.

### Limitation

Several limitations inevitably exist in this study. First, the inclusion and exclusion criteria for participants were not identified in this study, such as physical or psychological disorders, and these may have introduced bias into the analysis of the results. Second, the measurement of sleep in this study was subjective. Although the Chinese version of the PSQI is reliable in Chinese populations, studies have reported that people are unable to accurately report their duration of sleep (74). This may have over- or underestimated sleep duration in this study, thus introducing bias in the examination of the correlation between sleep and life satisfaction. Third, the present study was cross-sectional designed and this study design was incapable of verifying causal inferences. Consequently, future studies should identify inclusion and exclusion criteria for participants, objectively measure the outcome of sleep quality, and apply longitudinal and retrospective designs to provide more precise evidence. However, this study also has certain strengths in that it expands on related areas of research and can provide some insights into promoting the mental health of this particular group of high school students.

## Conclusion

This present study found that, among Chinese high school students, the prevalence of sleep problems was high, and the majority of students held moderate life satisfaction. Furthermore, there was a positive association between better sleep quality and greater life satisfaction, while sleep duration and daytime dysfunction significantly predicted life satisfaction among Chinese high school students. School and family interventions should increase the life satisfaction of high school students by improving the quality of their sleep, thereby promoting physical and mental health.

## Data availability statement

The original contributions presented in the study are included in the article/supplementary material, further inquiries can be directed to the corresponding author.

## Author contributions

YC and XH conceived the study and reviewed the first and final versions of the manuscript. YC collected and analyzed data and drafted the manuscript. ZY and YY assisted in revising the manuscript. All authors contributed to the article and agreed to the submitted version of the manuscript.
